# Is Organic Food Consumption Associated with Other Sustainable Food-Related Behaviors? Insights from a Survey in the Capital City of Poland

**DOI:** 10.3390/nu17132113

**Published:** 2025-06-25

**Authors:** Rita Góralska-Walczak, Lilliana Stefanovic, Renata Kazimierczak, Klaudia Kopczyńska, Lea Ellen Matthiessen, Carola Strassner, Benedetta Peronti, Patrizia Pugliese, Hamid El Bilali, Youssef Aboussaleh, Dominika Średnicka-Tober

**Affiliations:** 1 Department of Functional and Organic Food, Institute of Human Nutrition Sciences, Warsaw University of Life Sciences, Nowoursynowska 159c, 02-776 Warsaw, Poland; renata_kazimierczak@sggw.edu.pl (R.K.); klaudia_kopczynska@sggw.edu.pl (K.K.); dominika_srednicka-tober@sggw.edu.pl (D.Ś.-T.); 2 Section of Organic Food Quality, Faculty Organic Agricultural Sciences, University of Kassel, Nordbahnhofstrasse 1a, 34125 Witzenhausen, Germany; l.stefa@uni-kassel.de; 3 Department of Nutrition, Exercise, and Sports, University of Copenhagen, Rolighedsvej 26, 1172 Frederiksberg, Denmark; 4 Department of Food-Nutrition-Facilities, FH Münster University of Applied Sciences, Corrensstraße 25, 48149 Muenster, Germany; strassner@fh-muenster.de; 5 Council for Agricultural Research and Economics—Research Centre for Food and Nutrition (CREA—Food and Nutrition), Via Ardeatina, 12010 Rome, Italy; benedetta.peronti@crea.gov.it; 6 International Centre for Advanced Mediterranean Agronomic Studies (CIHEAM-Bari), 70010 Valenzano, Bari, Italy; pugliese@iamb.it (P.P.); elbilali@iamb.it (H.E.B.); 7 Department of Life Sciences, Faculty of Sciences, Ibn Tofail University, Kenitra 14000, Morocco; youssef.aboussaleh@uit.ac.ma

**Keywords:** organic, sustainable food system, sustainable diets, organic farming, Warsaw, household survey, SysOrg

## Abstract

**Background**: The current food system is unsustainable, making it essential to address the issue globally through adequate policies and sustainable development goals. The European Union aims to dedicate 25% of farmland to organic farming by 2030 to promote sustainable practices. Warsaw is the first Polish city working on an urban sustainable food policy; however, there is limited data on the sustainable food system (SFS) and organic sector available. **Objectives**: This research examines whether consumers in Warsaw who prefer organic food also display other sustainable characteristics and awareness, reflected in their food choices, dietary habits, and other food-related behaviors. **Methods**: A household survey (HHS) was conducted as part of the SysOrg project, focusing on evaluating the sustainability of food systems in Warsaw in the areas of diet and organic food. The clusters of respondents, grouped by the self-declared proportion of organic foods in their diets, were analyzed and compared, and in addition, correlation analyses of the share of organic food in diets and other sustainability parameters were performed. **Results**: The study of 449 respondents indicates that Warsaw is at an early stage of the organic transformation, with the largest group of respondents declaring a 1–10% share of organic products in their diet. There were significant differences in dietary choices, sustainability awareness, and food selection habits and motivations among various consumer groups depending on their organic food share. **Conclusions**: Overall, this study’s findings highlight a link between organic food consumption and certain sustainable behaviors, suggesting potential for organic consumers’ contribution to a sustainable transformation. The study offers valuable insights into the existing knowledge gap regarding the behaviors of organic and sustainable consumers in Warsaw. Furthermore, despite the non-random nature of the sample limiting the generalizability of findings, it serves as a preliminary resource for other European cities that are formulating food policies and incorporating Green Public Procurement (GPP) into their procurement processes, especially for municipalities within the Visegrad Group.

## 1. Introduction

The unsustainability of current food systems has resulted in significant environmental consequences, including land degradation, habitat and biodiversity loss, and the pollution of water, soil, and air [1]. Additionally, these systems contribute to negative health outcomes, such as malnutrition, obesity, and various non-communicable diseases [2,3]. In response to these and many other globally pressing issues, the United Nations (UN) has established the 2030 Agenda for Sustainable Development and the 17 Sustainable Development Goals (SDGs) as a roadmap for promoting global sustainability [4,5,6]. An immediate outcome of this political initiative has been the incorporation of the 2030 Agenda goals into the national policies of UN member states. The European Union (EU) has formulated a strategy through the adoption of the Green New Deal, which includes the Common Agricultural Policy [7,8]. This strategic framework aims to increase the proportion of organic agriculture to 25% by 2030 across the EU [9]. As an EU member state, Poland is mandated to “more than double its agricultural area under organic farming by 2030” [10]. While the organic sector is acknowledged for its contributions to the three dimensions of sustainable food systems (SFSs)—social, environmental, and economic [11]—it remains relatively minor in Poland, exhibiting stagnant dynamics. Currently, 4.4% of Poland’s total agricultural land is used for organic farming, in contrast to the EU average of 10.9% [12]. Furthermore, the distribution of organic farmland and operators within Poland is uneven [13].

Warsaw, the capital city of Poland, along with other postsocialist capitals in Central Europe that are part of the Visegrad Group (V4), such as Prague, Budapest, and Bratislava, aims to reach the economic and social standards of the wealthiest countries in Northern and Western Europe. In this year’s Financial Times ranking of “European Cities & Regions of the Future”, Warsaw is maintaining its leading position among Central and Eastern European cities, making its urban transformation a valuable example for other emerging cities [14,15]. It is also the first Polish city to sign and adopt the Milan Urban Food Policy Pact (MUFPP) [16]. The city is actively engaged in developing an urban food policy aimed at sustainable food management to ensure food security, reduce food waste, and promote sustainable consumption [17]. Sustainable food consumption (SFC) is a consumer-driven, holistic concept that encompasses the integrated implementation of sustainable patterns in food consumption and production while respecting the carrying capacities of natural ecosystems. This includes, among others, encouraging plant-rich diets, limiting meat consumption, and choosing sustainable agricultural practices [18,19]. In 2019, only 9% of adults in Poland reported consuming the recommended five portions of fruits and vegetables daily (below the EU average of 12%) [20], and in 2023, the daily consumption of fruits and vegetables reached about 280 g per capita (with the European Union average of 685 g per capita) [21]. Concurrently, meat consumption has steadily increased, reaching 79.2 kg per person annually by 2022 [22,23]. Organic consumption, which constitutes a part of SFC [24], remains relatively low in Poland, with individuals spending on average EUR 8 per capita annually on certified organic food. In comparison, in Switzerland, the country with the highest per capita expenditures on organic food, it is EUR 468 per person per year—a large distinction, even when factoring in higher earnings [12]. The organic market, being a niche market, is similar across all the V4 group countries, with comparable consumer behaviors regarding the purchase of organic food [15]. However, the situation is dynamic, as policies are evolving and cities are establishing strategic sustainable frameworks, e.g., sustainable public procurement [25].

The SysOrg project, titled “Organic Agro-Food Systems as Models for Sustainable Food Systems in Europe and Northern Africa”, aims to characterize and analyze five specific territorial cases, namely the Warsaw municipality in Poland, the North Hessia region in Germany, the Cilento bio-district in Italy, the Kenitra province in Morocco, and the Copenhagen municipality in Denmark, with the aim to identify pathways for enhancing sustainable and organic consumption and food production. Next to perspectives such as food system transitions, sustainable and healthy diets, and food waste problems, it investigates key areas related to organic food and farming–agricultural systems that have tangible contributions to environmental protection and resource conservation through, i.e., water protection, carbon storage, ecosystem service resilience, biodiversity protection, and GHG emissions reduction [26,27,28].

As for Warsaw, there is a dearth of data regarding the residents’ sustainable organic consumption and behaviors. This paper seeks to provide a preliminary overview of organic consumption patterns based on the household survey (HHS) study sample in the capital city. It also aims to explore whether individuals with higher levels of organic consumption demonstrate higher sustainability awareness and more sustainable behaviors regarding their dietary choices compared to those who do not engage in organic consumption.

## 2. Materials and Methods

### 2.1. Data Collection: Household Survey

The household survey applied in this study was developed by the international SysOrg project consortium [29] to collect comprehensive data on three key sustainability perspectives addressed in the project, which are diet, organic food consumption, and food waste in different SysOrg case territories. The present study focuses on the territory of Warsaw and takes into consideration a range of HHS aspects, including socio-demographic information, the frequency of food consumption (including organic food), the proportion of organic foods in participants’ diets, shopping behaviors, perceptions of sustainability and organic products, and food choice determinants. Certain questions were derived from previously validated questionnaires [30,31,32]. The selected questions from the survey are reported in the Supplementary Materials (Table S1). The HHS, administered using Lime Survey© (https://www.limesurvey.org/) was distributed through the river sampling methodology (a non-probabilistic approach to recruiting respondents online) [33] by diverse social media channels (e.g., Facebook, Instagram) and online sites (e.g., Municipality of Warsaw). To reach respondents from 18 districts of Warsaw, the survey was circulated through the online communication channels of different neighborhoods of the city. Altogether, 484 adults (>18 years old) completed the survey, and after data cleaning (a process aiming to develop one standardized dataset for the results of the SysOrg household survey for all 5 case study territories excluding respondents speeding and with incomplete answers), the responses of 449 Warsaw residents were included in further analyses. The characteristics of the included respondents are presented in Appendix A. Previous research has displayed additional characteristics of the study group [29].

Data collection was conducted in compliance with the European Commission’s General Data Protection Regulation (679/2016), and the study followed the ethical guidelines set forth by the Declaration of Helsinki [34]. All participants provided informed consent for the use of their responses in the anonymously completed HHS. The research protocol received approval from the Scientific Research Ethics Committee at the Institute of Human Nutrition Sciences of the Warsaw University of Life Sciences (approval number: 50/2021; date: 15 November 2021).

At the outset of the survey, participants were instructed to ensure that it was completed by the adult individual primarily responsible for food preparation and purchasing within the household. Analytical methods were employed to explore associations between the self-reported share of organic food in respondents’ diets (0%, 1–10%, 11–25%, 26–50%, 51–75%, 76–99%, and 100%) and the responses to questions pertinent to healthy and sustainable behaviors. Considering the distribution of the surveyed population in terms of the declared share of organic food in the diet, some results were presented in four groups, namely the 0%, 1–10%, 11–25%, and 26–100% groups.

### 2.2. Data Analysis

The following descriptive statistics were utilized to characterize the study sample: the number of observations, percentages, and means. Pearson’s chi-squared test was applied to evaluate the relationships between the qualitative variables. Associations between the quantitative variables were analyzed using Spearman correlation analysis. A *p*-value of less than 0.05 was considered significant for all tests conducted. Factor analysis with a principal component extraction method was used to obtain factors defining certain dietary patterns (DPs). This statistical method helps uncover dietary patterns based on the consumption frequencies of certain food groups. In this case, it allowed us to identify distinct eating behavior profiles—such as plant-based or meat-heavy diets—based on how respondents reported their intake of various food groups. The factors were subjected to a varimax-normalized rotation. The number of factors was selected based on the following criteria: components with an eigenvalue of 1 or more, a scatter plot, and the interpretability of the factors. The selection of factors was confirmed using the Kaiser–Meyer–Olkin (KMO) measure of sampling adequacy and Bartlett’s sphericity test. Bartlett’s test value was *p* < 0.001, and the KMO value for DPs was 0.780. Factor loadings of 0.5 or higher were used to identify factor components of certain DPs. The statistical analyses were performed using Statistica software version 13.3 PL (StatSoft Inc., Tulsa, OK, USA; StatSoft, Krakow, Poland).

## 3. Results and Discussion

### 3.1. Organic Consumption in Warsaw—Overview

**Figure 1:**
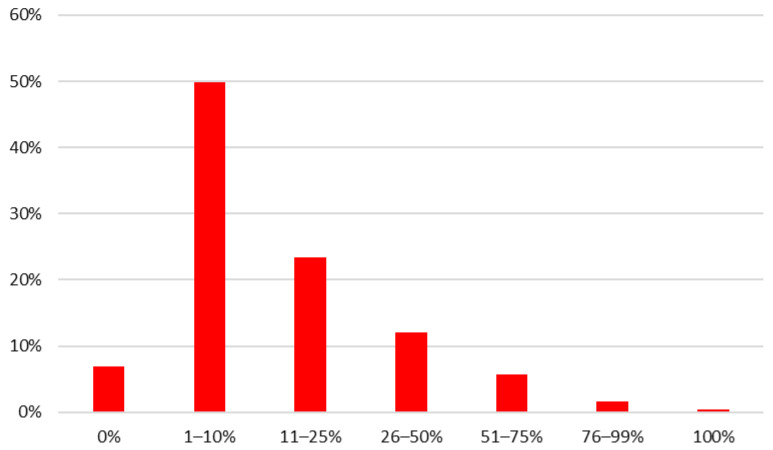
Answers to the household survey question: “What percentage, by volume, of the foods you eat is organic?” by respondents in Warsaw. N = 449.

This study’s findings, based on the 449 respondents, indicate that Warsaw is still at an early stage of developing its organic food market. According to the HHS, 50% of respondents classified themselves in the low-organic consumption group (1–10%), while 7% reported consuming no organic products at all (0%). In contrast, 8% indicated high (>50%)-organic consumption (Figure 1). The low consumption of organic food in Poland has also been previously documented through the country’s research [35].

**Figure 2:**
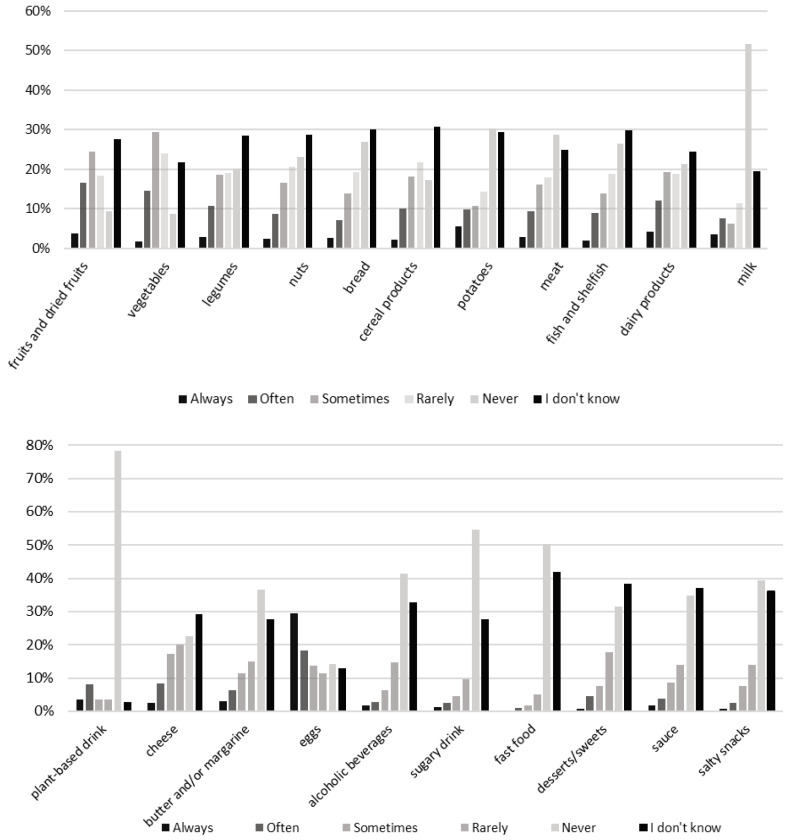
Answers to the household survey question: “How often are the [product group] you eat certified organic?” by respondents in Warsaw. N = 449.

In the survey, about 20% of respondents indicated that they always or often choose organic options for vegetables, fruits, legumes, and eggs—consequently, these products are among the most frequently consumed organic items. In contrast, 78% and 53% of respondents reported that they never select organically produced plant-based drinks and milk, and 25–30% of respondents declared never opting for common staple foods, such as organic potatoes, meats, fish, or bread. Additionally, the less healthy food categories seemed to be more often consumed as conventional products, such as alcohol, sugary drinks, fast food, desserts, sweets, and salty snacks (36–55% indicated that they never opt for organic options) (see Figure 2). The preference for organic vegetables and fruits, as well as eggs, is also supported by the Farma Świętokrzyska report from 2021 [36] and further supported by findings from a study by Wojciechowska-Solis and Śmiglak-Krajewska in 2021 [37], particularly in the case of organic vegetables. Milk alternatives represent a category of products that may be unfamiliar to Polish consumers, often accompanied by less favorable associations [38,39]. Furthermore, there is a deficiency in communication and information regarding the various options available on the market [38,39], and as a result, the majority of consumers may not know about organic plant-based drink selections. As for milk, in Poland, the limited number of organic milk processors hinders the growth of the organic dairy sector [40], translating into limited market offerings, which may explain the lack of preference for organic choices in this study. However, the situation is dynamically changing in this context, since big dairy market players have recently started developing organic production lines. The low consumption of organic fast foods, sweets, and alcohol may also be connected with the low availability of organic options of these food categories in Poland [41]. Previous Danish research shows that the majority of fast-food customers want healthier and more sustainable fast food menu options; however, taste and price remain the major drivers for actual purchase decisions within this food category [42].

### 3.2. Associations Between Organic and Sustainable Food-Related Behaviors in Warsaw

#### 3.2.1. Dietary Behavior

In Poland, as well as in other countries, according to the current dietary recommendations [3,43], it is advisable to reduce red meat consumption and use more plant-based protein sources, including legumes and nuts, as well as incorporating fish and eggs as an alternative [44,45]. This approach is consistent with the recommendations outlined in the Planetary Health Diet (PHD) [46] to increase the consumption of healthy foods, such as nuts, fruits, vegetables, and legumes, by more than 100%, while halving meat, processed meat, and sugar consumption for both health and environmental reasons [2].

**Figure 3:**
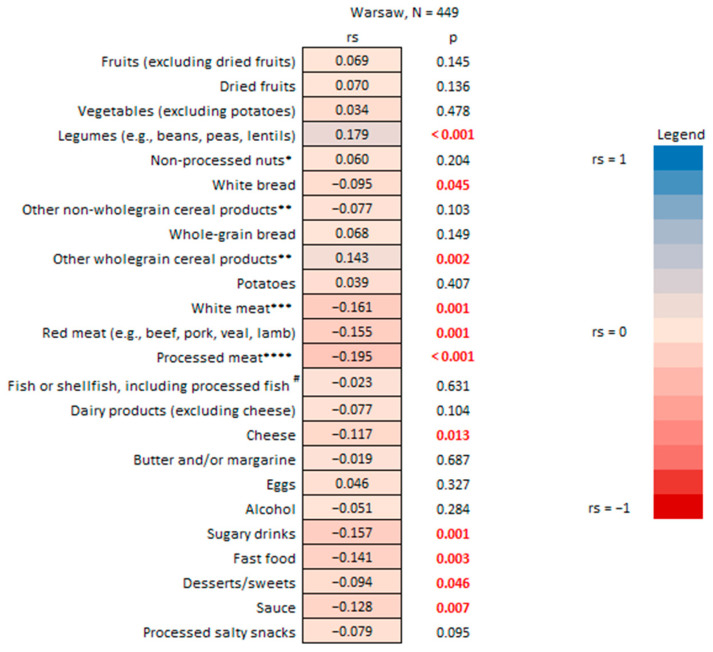
The organic food consumed vs. frequency of consumption of selected food product groups by household survey respondents in Warsaw. * Including peanuts (e.g., unsalted, non-roasted, not sugar-coated); ** e.g., pasta, rice; *** e.g., rabbit, chicken, turkey, other poultry; **** e.g., cured ham and turkey, salami; # e.g., canned tuna, smoked salmon. R-Spearman correlation analysis (*p* < 0.05).

**Figure 4:**
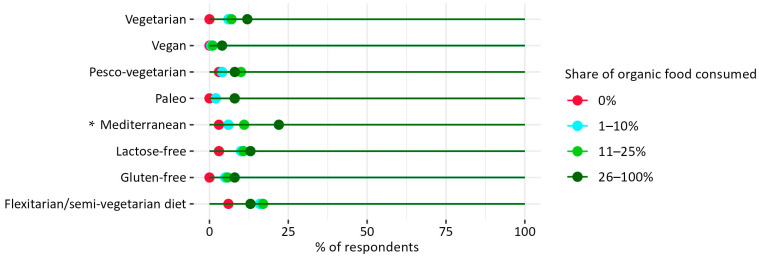
Percentage of organic food consumed vs. following a special diet by household survey respondents in Warsaw. * Pearson’s chi-squared test results for the overall relationship between variables (Chi^2^ = 23.27; *p* < 0.001). N = 449.

According to the findings of the present study, Warsaw HHS respondents who consume organic food tend to demonstrate a trend for healthier dietary patterns and more sustainable eating practices (Figure 3 and Figure 4). The self-reported organic food consumption was positively correlated with the frequency of consumption of legumes and whole grains. Conversely, a more limited organic food share in diets was associated with a higher frequency of meat (white, red, processed), white bread, and dessert/sweet consumption. Similarly, a negative association was noted between the consumption of organic food vs. sugary drinks and fast food—the food groups linked to a higher risk of adverse health outcomes, particularly cardiometabolic issues, and mortality [47]. The healthier dietary behavior of organic compared to non-organic consumers was also confirmed by a Polish study on diet quality indicators and organic food consumption in a group of mothers of young children [48].

Respondents from the group that consumed a higher proportion of organic food (26–100%) reported more frequently that they follow a Mediterranean diet (Figure 4). This dietary pattern is recognized as beneficial for both health and environmental sustainability, especially when organic products are utilized, as they are known for being less frequently contaminated with detectable pesticide residues [49,50]. Furthermore, this diet aligns with the definition of sustainable diets proposed by the Food and Agriculture Organization of the United Nations (FAO) [51] and the double food pyramid [52]. These findings are partially corroborated by recent research conducted in Spain, associating organic consumption with Mediterranean dietary patterns [53,54].

Notably, a greater number of individuals within the group reporting higher organic food share in their diet also practice vegan and vegetarian diets, which are acknowledged for their lower environmental impacts [53].

Furthermore, to identify dietary patterns (DPs), a factor analysis with a principal component extraction method was performed. The seven eating behavior patterns were identified (designated as DP1-DP7), characterized by the following factor loadings: “DP1”—characterized by the consumption of meat and eggs; “DP2”—fruits, vegetables, legumes, and unprocessed food; “DP3”—processed food and fast food; “DP4”—dairy, including cheese and butter; “DP5”—white bread and non-whole grain cereal products (e.g., pasta, rice); “DP6”—whole grain bread and other whole grain cereal products (e.g., pasta, rice); and “DP7”—alcohol (Table 1).

The research reveals a positive statistically significant association between a healthier, environmentally friendly, plant-based dietary pattern (DP2)—which prioritizes the consumption of fruits, vegetables, and legumes—and higher levels of organic food consumption (Table 1). Conversely, a negative correlation was identified between organic food consumption and primarily animal-based patterns such as DP1 (meat and eggs), DP4 (dairy, including cheese and butter), and DP3 (processed and fast food products). This is supported by a French study showing that the dietary share of plant-based foods increased with the contribution of organic foods to the diet, whereas a reverse trend was identified for meats and processed meats, dairy products, cookies, fast-food, and soda [55]. Furthermore, in the same study, higher organic food consumption was associated with better adherence to NDGs, which aligns with findings from other studies from Denmark [56].

#### 3.2.2. Sustainability Awareness, Food Choice Determinants, Associations with Organic Food, and Shopping Places

Previous studies have exposed that sustainability awareness is a significant motivator for purchasing organic foods, with concerns about pesticide use, environmental degradation, and health implications driving consumer behavior [57,58].

**Figure 5:**
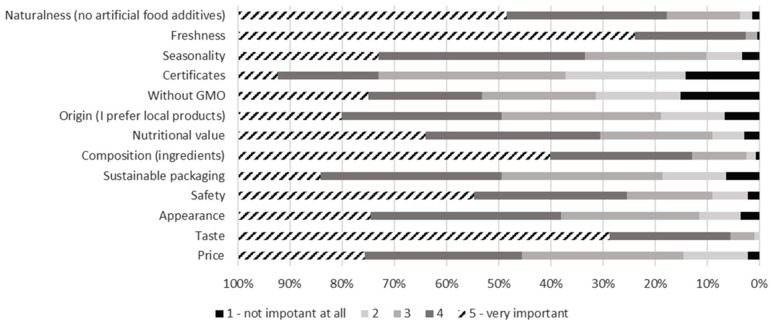
Answers to the household survey question: “How important are the following attributes for your food choices?” by respondents in Warsaw. Grading from 1—not important at all, to 5—very important. N = 449.

In addition, the other studies show that the capital city of Warsaw, which possesses the largest and most competitive market in Poland [59], alongside one of the highest per capita incomes in the country [60], reveals specific consumer behavior patterns. In comparison to other cities in Poland, 64% of Warsaw residents spend more than the national average on food shopping, and the city has experienced the lowest impact of inflation on food expenditures [61]. The same report [55] highlights that the inclination to opt for higher-quality products is a contributing factor to this trend. In fact, freshness, taste, and composition, alongside naturalness, no GMOs, and safety, were identified as the most significant factors influencing food purchasing decisions among Warsaw consumers in this study (Figure 5).

This is corroborated by findings from another Polish study [62] indicating that freshness and ingredient quality are paramount considerations for food shopping in the capital. However, the Report on the Study of Shopping and Eating Habits of Warsaw Residents in the Context of Food Waste [63] emphasizes the importance of price as a determining factor in food choice. In the context of this study, the attribute of price was deemed highly important by only 5% of respondents. Conversely, a substantial 25% of participants identified it as the attribute they considered not important at all (Figure 5). It is worth mentioning that the highest income group also represented the largest portion of respondents in the current survey, accounting for 24% (see Appendix A).

**Figure 6:**
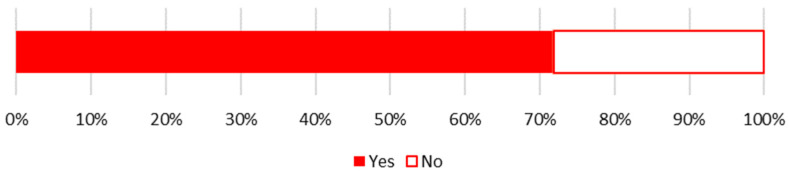
Answers to the household survey question: “Do you obtain food from sources other than supermarkets, such as farmers’ markets, food box programs, community-supported agriculture (CSA)?” by respondents in Warsaw. N = 449.

**Figure 7:**
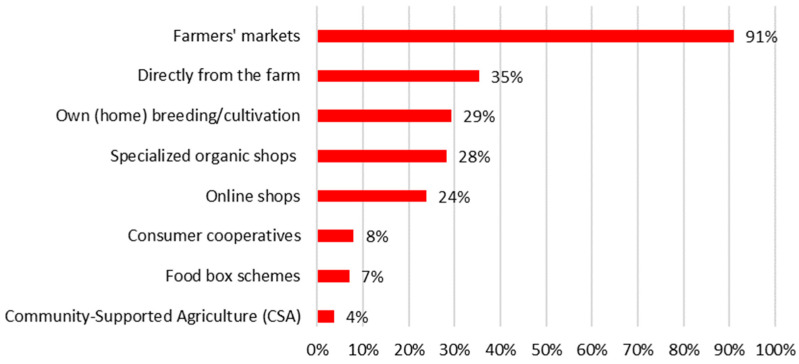
Answers to the household survey question: “In which of the initiatives and alternative places do you source your food?” by respondents in Warsaw. N = 323 (only those respondents who admitted that they obtain food from sources other than supermarkets).

The three attributes referenced above (freshness, taste, and composition) may elucidate why over 70% of respondents declared to be engaged in sustainable shopping behaviors (Figure 6). Farmers’ markets emerge as the most favored source for procuring food outside of supermarkets, with 91% of those respondents indicating this preference (Figure 7).

Engaging in more sustainable shopping practices, particularly through the use of farmers’ markets, has been shown to be associated with a growing dissatisfaction with globalized food supply systems. This dissatisfaction often stems from concerns regarding the traceability and transparency of production processes, as well as perceived deficiencies in food quality [64]. It is also motivated by the related environmental concerns of city-dwellers, like ecological footprint and food miles—the distance food travels from production to the consumer [65].

**Figure 8:**
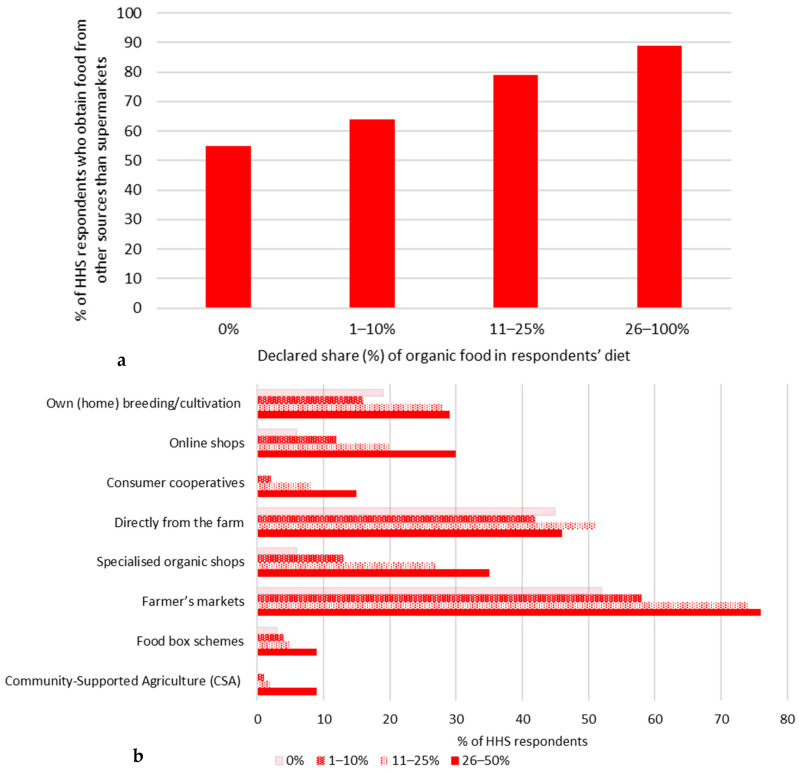
(**a**) Answers to the household survey question: “Do you obtain food from sources other than supermarkets, such as farmers’ markets, food box programs, community-supported agriculture (CSA)?” vs. declared share of organic food (%). (**b**) Answers to the household survey question: “In which of the initiatives and alternative places do you source your food?” vs. declared share of organic food. N = 323 (only those respondents who admitted that they obtain food from sources other than supermarkets).

This research indicates that the group consuming the highest percentage of organic food tends to procure their provisions from sources other than supermarkets more frequently than those with lower organic consumption (Figure 8a). Moreover, the respondents from this group indicate more often that they acquire their products from a variety of the alternative venues mentioned, especially farmers markets; however, respondents declaring 11–25% share of organic food in their diet tend to be more actively involved in direct farm purchases (Figure 8b).

**Figure 9:**
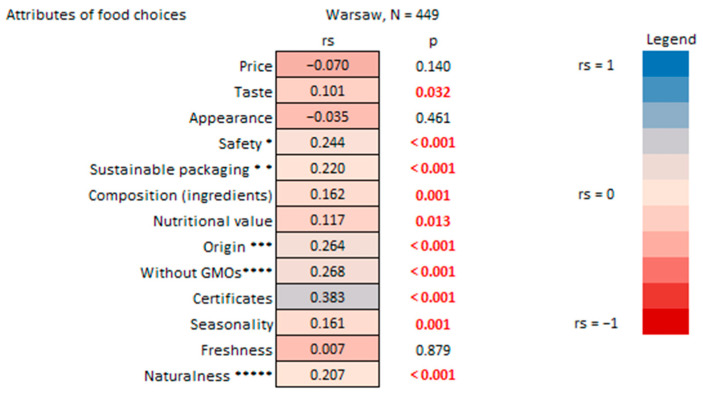
Percentage of organic food consumed by household survey respondents in Warsaw vs. attributes of their food choices. * e.g., pathogens, pesticide residue; ** e.g., biodegradable, reusable, non-plastic; *** I prefer local products; **** genetically modified organisms; ***** no artificial food additives. R-Spearman correlation analysis (*p* < 0.05).

Moreover, this study reveals that higher levels of organic food consumption are linked to a greater focus on sustainable and healthy attributes in food selection. A statistically significant positive correlation was identified between the reported percentage of organic food in respondents’ diets and the importance of certifications for their food choices (Figure 9). Furthermore, there were significant positive correlations between organic consumption and preferences for “without GMO” products, as well as local products. While the environmental concerns associated with GMOs are widely discussed and increasingly acknowledged [66], choosing local products can bolster local economies and encourage sustainable agricultural practices [67]. Additionally, there was a positive correlation between organic consumption and sustainable packaging practices and food seasonality.

The importance of food safety (cf. pathogens, pesticide residues) as an attribute impacting food choices also demonstrated a statistically significant positive correlation with the percentage of organic food consumed (Figure 9). Additionally, nutritional value, naturalness (no artificial food additives), and composition (ingredients) also proved a statistically significant positive correlation with organic consumption.

**Figure 10:**
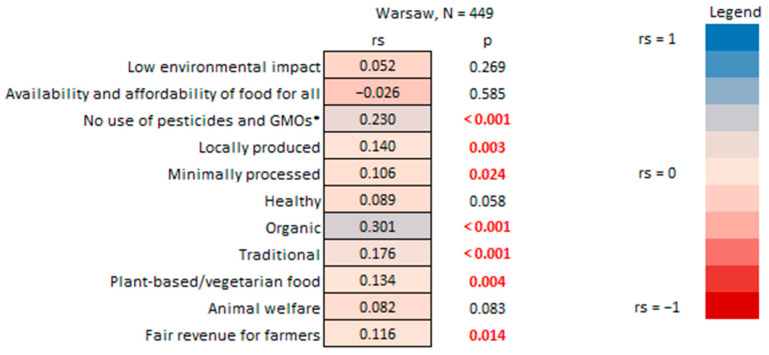
Answers to the household survey question: “How important are these aspects when you think about “sustainable” food?” vs. the percentage of organic food consumed by household survey respondents in Warsaw. * GMOs—genetically modified organisms. R-Spearman correlation analysis (*p* < 0.05).

Correspondingly, respondents consuming more organic food rated significantly higher the importance of aspects of “sustainable food” such as food being organic, with no use of pesticides and GMOs, as well as food that is traditional, locally produced, minimally processed, healthy, and plant-based and provides fair revenue for farmers (Figure 10).

The two abovementioned results (Figure 9 and Figure 10) align with findings from another Polish study that explored the connection between higher environmental awareness and the inclination to purchase organic products, highlighting concerns for the environment, the absence of harmful substances in food production, and a low level of processing [68].

**Figure 11:**
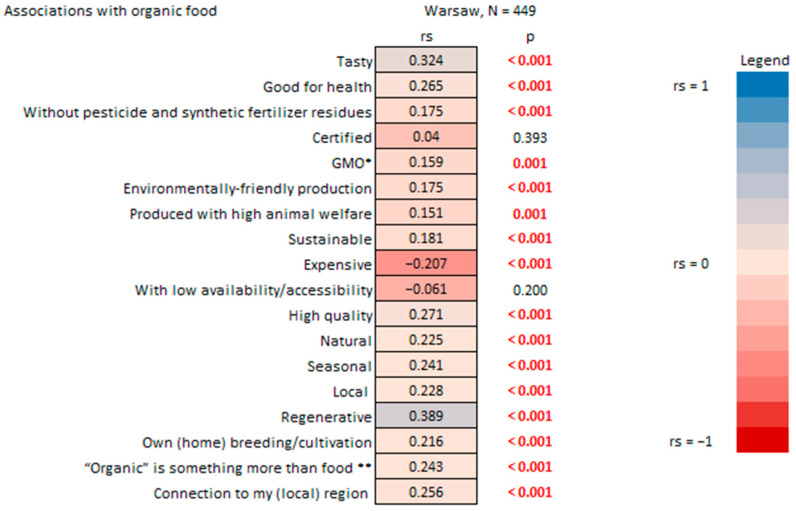
Percentage of organic food consumed by household survey respondents in Warsaw vs. associations with organic food. * Genetically modified organisms; ** it is a lifestyle, philosophy, and it transports values. R-Spearman correlation analysis (*p* < 0.05).

Moreover, a positive correlation was identified between organic consumption and most of the encouraging associations with organic food mentioned in the HHS (Figure 11). The understanding of organic food for organic consumers is associated positively with terms that refer to something more than just a dietary choice. It is associated with “regenerative” aspects, which can be connected to multiple dimensions of sustainability practices [69], but it is also linked to a connotation representing a lifestyle and philosophy that embodies specific values (Figure 11). This indicates that “organic consumers” in Warsaw tend to embrace a more holistic view of the organic food system. This is confirmed by another study that shows that “organic consumers” adhere to more sustainable consumption principles [70] and this “values view” in food systems, according to the research by Varzakas and Antoniadou [24], is important to promote and prioritize sustainability.

#### 3.2.3. Willingness to Change Food Habits to More Sustainable Ones

**Figure 12:**
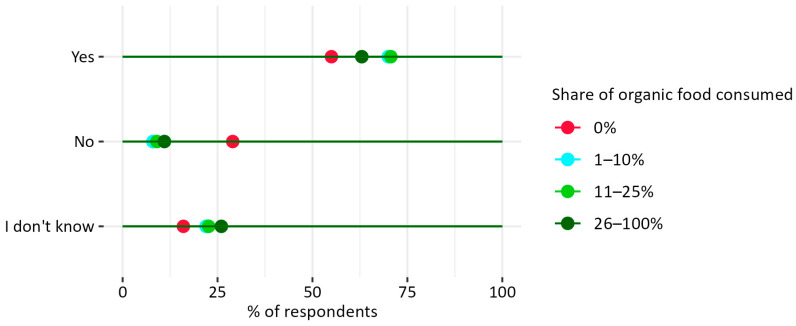
Answers to the question: “Are you ready to change your food habits into more sustainable ones” vs. self-reported percentage of organic food in the diet of respondents in Warsaw. Pearson’s chi-squared test results for the overall relationship between variables (Chi^2^ = 19.85, *p* = 0.069). N = 449.

**Figure 13:**
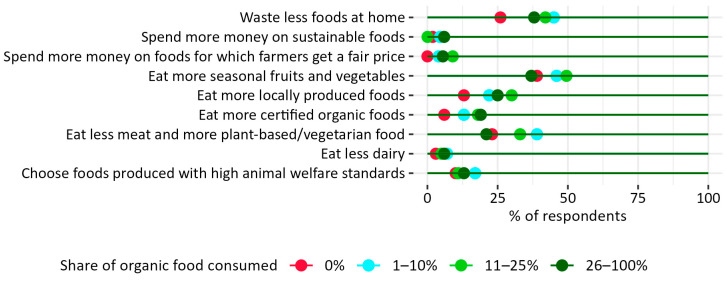
Self-declared percentage of organic food in the respondents’ diet vs. the declared actions that they are ready to undertake to change their food habits into more sustainable ones. N = 254 (respondents who admitted that they are ready to change their diets to more sustainable ones).

The connection between nascent sustainability awareness and organic consumption may be the reason why 70% of respondents who consume 1–10% of organic food are reporting their willingness to change their eating habits to be more sustainable (Figure 12) and 19% of them are willing to eat more food with an organic certificate (Figure 13). The results showing a statistical trend may signal an increase in the number of organic consumers associated with other sustainable changes in the food system—more than 55% of the members of this group are also willing to eat less meat and more plant-based foods, opt for products with high animal welfare standards, and waste less food (Figure 13).

## 4. Conclusions

Although Warsaw is at an early stage of a sustainable urban food system transformation, its citizens are showing readiness to embrace this change. They prefer higher-quality food products and most likely prioritize quality over price in their shopping habits, frequently engaging in alternative food purchasing practices. HHS respondents with higher organic consumption tend to adopt healthier and more sustainable eating habits. Additionally, sustainable and healthy aspects are important to them when selecting food, embracing a values-driven perspective on the food system. This mindset is important for promoting ethical practices, enhancing transparency, restructuring economic frameworks, and striving for a more sustainable and equitable food system. Moreover, there is significant potential for a sustainability transition among the group that currently consumes less organic food (around 1–10%), with a majority indicating a willingness to adjust their eating habits toward more sustainable options. Consequently, it should be a priority for the city engaged in food policy to raise awareness about sustainability and encourage sustainable and organic consumption, adjusting actions to the main actors, for example, encouraging organic farmers to participate in farmers’ markets and promoting the most popular organic products like vegetables, fruits, and eggs. When considering public procurement, the above-mentioned organic products should be included, being the most preferred. Additionally, this research demonstrates that highlighting nutrition and health benefits can effectively enhance the marketing of organic products.

These results are opening up promising opportunities in regions where organic consumption has yet to gain traction. As such, Warsaw serves as a valuable case study for other regions in Central Europe, particularly within the V4 group cities like Budapest and Prague that have similar levels of organic consumption. This is especially relevant in addressing contemporary challenges, such as implementing Green Public Procurement (GPP) in public catering and developing sustainable urban food policies in all of these urban realities.

The approach used in this study has its strengths but also limitations. The primary limitation of this study was the implementation of the “river” sampling methodology—a non-probabilistic approach to participant selection, which limits the generalizability of findings given the non-random nature of the sample. Despite this constraint, the methodology enabled us to engage with a substantial number of participants, thereby facilitating data collection. The primary strength of this study lies in its contribution to filling the existing knowledge gap regarding the links between organic food and other sustainable consumer food-related behaviors in Warsaw, providing valuable data for the development of urban food policy, in line with the Sustainable Development Goals (SDGs) and the European Green Deal policies.

## Figures and Tables

**Table 1:** The association between organic food consumption and the identified dietary patterns (DPs) of household survey respondents in Warsaw. N = 449.

Variables	*rs*	*p* *
DP 1 (meat and eggs)	−0.148	0.002
DP 2 (fruits, vegetables, legumes, not processed)	0.143	0.002
DP 3 (processed food and fast food)	−0.123	0.009
DP 4 (dairy, including cheese and butter)	−0.108	0.022
DP 5 (white bread and non-whole grain cereal products)	−0.055	0.241
DP 6 (whole bread and whole grain cereal products)	0.067	0.157
DP 7 (alcohol)	0.071	0.132

* R-Spearman correlation analysis (*p* < 0.05).

## Data Availability

The original contributions presented in this study are included in the article. Further inquiries can be directed to the corresponding author.

## Supplementary Materials

The following supporting information can be downloaded at: https://www.mdpi.com/article/10.3390/nu17132113/s1, Table S1: Household survey—selected questions used for this publication; Table S2: Frequency of choosing organic products by household survey respondents in Warsaw (% of respondents); Table S3: Answers to the household survey question: “How important are the following attributes for your food choices?”.

## Abbreviations

The following abbreviations are used in this manuscript:

CSA	Community-supported agriculture
DP	Dietary pattern
FAO	The Food and Agriculture Organization of the United Nations
FS	Food system
GMOs	Genetically modified organisms
GPP	Green Public Procurement
HHS	Household survey
KMO	Kaiser–Meyer–Olkin
MUFPP	Urban Food Policy Pact
NDGs	National dietary guidelines
PHD	Planetary Health Diet
SFS	Sustainable food system
SDGs	Sustainable Development Goals
SFC	Sustainable food consumption
UN	United Nations
V4	Visegrad Group
WHO	The World Health Organization

## Appendix A

**Table A1:** Characteristics of the household survey study group.

Variables	N = 449	%
Gender		
Male	76	16.9
Female	373	83.1
Age in years (M ± SD)	38.6 ± 13.2	
Age in categories		
18–26	97	21.6
27–35	95	21.2
36–44	119	26.5
45–53	72	16.0
54 or more	66	14.7
Level of education		
Secondary or apprenticeship	91	20.3
Bachelor’s degree or equivalent level	68	15.1
Master’s degree or equivalent level	248	55.2
Doctoral studies and/or higher	42	9.4
Disposable net household income per year (PLN)		
I prefer not to answer	109	24.3
Up to 30,000 (lower)	34	7.6
30,001–45,000 (low)	31	6.9
45,001–60,000 (medium-low)	37	8.2
60,001–77,000 (medium)	33	7.4
77,001–96,000 (medium-high)	45	10.0
96,001–120,000 (high)	53	11.8
More than 120,000 (highest)	107	23.8
