# Supplementary material for: Is Organic Food Consumption Associated with Other Sustainable Food-Related Behaviors? Insights from a Survey in the Capital City of Poland
Source: Nutrients. 2025 Jun 25;17(13):2113. doi: 10.3390/nu17132113 (PMC12251464; doi:10.3390/nu17132113)
Supplement: Supplementary file 1 [file nutrients-17-02113-s001.zip › nutrients-3672054-supplementary.pdf]

nutrients

Supplementary Materials

Table S1. Household Survey – selected questions used for this publication

Household Survey - Selected questions used for this publication

Organic agro-food systems as models for sustainable food systems in Europe and Northern Africa

Online Title:
Household survey of diet, organic food consumption and food waste

Consent form
You are being invited to participate in a research study entitled "*Organic agro- food systems as models for sustainable food systems in Europe and Northern Africa*" (SysOrg) that is being conducted by [NAME of steering committee member], [POSITION, e.g. Professor] at [Institution]. This is a collaborative research with partners in Denmark, Italy, Poland, Germany and Morocco.
The questionnaire should take around 30 minutes to complete. Your participation in this study is voluntary. At any time and for any reason, you can refuse to answer a question or stop filling out the questionnaire.
If you agree to participate, you will remain completely anonymous, which means that none of the researchers who work with the analysis of the responses can identify who you are or have access to your personal data. The type of data assessed in this survey does not contain any personal information such as your name and is therefore

not subject to the European General Data Protection Regulation (GDPR). The answers will be used exclusively for statistical purposes in compliance with current national legislation. The European project partners - University of Copenhagen, (DK), Warsaw University of Life Sciences, (PL), Council for agricultural research and economics - CREA, (IT), FH Münster University of Applied Sciences, University of Kassel (DE), and International Centre for Advanced Mediterranean Agronomic Studies – Mediterranean Agronomic Institute of Bari (CIHEAM-Bari), (IT) - will process the results of this study. The data collected will be used for survey reports and for scientific publications.

If you have any questions about this research, you can contact us by sending an email to [NAME].

Consent

The study described above has been explained to me. I understand that future questions I may have about the research will be answered promptly by the investigators listed above.

By selecting "next", the subject certifies that he/she is at least 18 years of age.

☐Yes, I agree to participate in this study

☐No*, I do not agree to participate in this study

Sociodemographic information

The questionnaire should be filled out by the person primarily responsible for preparing and purchasing food in your household.

**Do you live in this territory [TERRITORY NAME]?**	
☐Yes	☐No*
**Which municipality/administrative part of the city/region (district) do you live in?**	
Name: ______________________________ Zip Code: _________________________________	
**Your age (in years)**	

**Your gender****			
☐Female	☐Male	☐No pronoun	☐ I prefer not to answer
**Your level of education****			
☐No formal education	☐Primary education (1-4 years)	☐ Lower secondary education (5-10 years)	☐ Upper secondary education (10-13 years)
☐Apprenticeship (2-3 years)	☐Bachelor's degree or equivalent level (3 years)	☐Master's degree or equivalent level (e.g. Diploma) (3+2 years)	☐ Doctoral studies (PhD) and/or higher
**Household members**			
Total number of household members			
Age in Years	Female Members	Male Members	Other
< 1			
1-9			
10-17			
Adults (≥18)			
**Disposable Net Household Income (in Euro) per year****			
☐Up to 18.000	☐18.001-27.000	☐27.001-36.000	☐36.001-46.000
☐46.001-57.000	57.001-72.000	☐More than 72.000	☐I prefer not to answer
**How much of your net monthly household income is approximately spent on food purchase per month?**			

☐<10%	☐10-25%	☐26-50%	☐>50%

What percentage, by volume, of the foods you eat is ORGANIC?

ORGANIC food - produced according to standards for organic farming, ie. without synthetic pesticides, synthetic fertilizers, genetically modified organisms (GMOs), synthetic additives, and with the least possible processing (e.g. no radiation); organic production is controlled and products are certified by control bodies;
in the EU organic food products are labelled with "Euro-leaf".

☐0%	☐1-10%	☐11-25%	☐26-50%	☐51-75%	☐76-99%	☐100%

How often do you eat fruits (excluding dried fruits)? (*Tick one box*)

☐Never	☐ Less than once a month	☐1-3 times per month	☐Once a week	☐2-4 times per week	☐5-6 times per week	☐Once a day	☐2-3 times per day	☐4-5 times per day	☐Every time I eat

How often do you eat dried fruits? (*Tick one box*)

☐Never	☐ Less than once a month	☐1-3 times per month	☐Once a week	☐2-4 times per week	☐5-6 times per week	☐Once a day	☐2-3 times per day	☐4-5 times per day	☐Every time I eat

How often are the fruits and dried fruits you eat certified organic?*** (*Tick one box*)

☐Always	☐Often	☐Sometimes	☐Rarely	☐Never	☐I don't know

How often do you eat vegetables (excluding potatoes)? (*Tick one box*)

☐Never	☐ Less than once a month	☐1-3 times per month	☐Once a week	☐2-4 times per week	☐5-6 times per week	☐Once a day	☐2-3 times per day	☐4-5 times per day	☐Every time I eat

How often are the vegetables you eat certified organic?*** (*Tick one box*)

☐Always	☐Often	☐Sometimes	☐Rarely	☐Never	☐I don't know

How often do you eat legumes (e.g. beans, peas, lentils)? (*Tick one box*)

☐Never	☐ Less than once a month	☐1-3 times per month	☐Once a week	☐2-4 times per week	☐5-6 times per week	☐Once a day	☐2-3 times per day	☐4-5 times per day	☐Every time I eat

How often are the legumes you eat certified organic?*** (*Tick one box*)

☐Always	☐Often	☐Sometimes	☐Rarely	☐Never	☐I don't know

How often do you eat non-processed nuts, including peanuts (e.g. unsalted, non-roasted, not sugar-coated)? (*Tick one box*)

☐Never	☐ Less than once a month	☐1-3 times per month	☐Once a week	☐2-4 times	☐5-6 times per week	☐Once a day	☐2-3 times per day	☐4-5 times per day	☐Every time I eat

				per week					
**How often are the nuts you eat certified organic?***** (*Tick one box*)									
☐Always		☐Often	☐Sometimes		☐Rarely		☐Never		☐I don't know
**How often do you eat whole-grain bread?** (*Tick one box*)									
☐Never	☐ Less than once a month	☐1-3 times per month	☐Once a week	☐2-4 times per week	☐5-6 times per week	☐Once a day	☐2-3 times per day	☐4-5 times per day	☐Every time I eat
**How often do you eat white bread?** (*Tick one box*)									
☐Never	☐ Less than once a month	☐1-3 times per month	☐Once a week	☐2-4 times per week	☐5-6 times per week	☐Once a day	☐2-3 times per day	☐4-5 times per day	☐Every time I eat
**How often is the bread you eat certified organic?***** (*Tick one box*)									
☐Always		☐Often	☐Sometimes		☐Rarely		☐Never		☐I don't know
**How often do you eat other non-whole grain cereal products (e.g. pasta, rice)??** (*Tick one box*)									
☐Never	☐ Less than once a month	☐1-3 times per month	☐Once a week	☐2-4 times per week	☐5-6 times per week	☐Once a day	☐2-3 times per day	☐4-5 times per day	☐Every time I eat
**How often do you eat other whole grain cereal products (e.g. pasta, rice)?** (*Tick one box*)									
☐Never	☐ Less than once a month	☐1-3 times per month	☐Once a week	☐2-4 times per week	☐5-6 times per week	☐Once a day	☐2-3 times per day	☐4-5 times per day	☐Every time I eat
**How often are the cereal products you eat certified organic?***** (*Tick one box*)									
☐Always		☐Often	☐Sometimes		☐Rarely		☐Never		☐I don't know

**How often do you eat potatoes?** (*Tick one box*)									
☐Never	☐ Less than once a month	☐1-3 times per month	☐Once a week	☐2-4 times per week	☐5-6 times per week	☐Once a day	☐2-3 times per day	☐4-5 times per day	☐Every time I eat

**How often are the potatoes you eat certified organic?***** (*Tick one box*)					
☐ Always	☐Often	☐Sometimes	☐Rarely	☐Never	☐I don't know

**How often do you eat white meat (e.g. rabbit, chicken, turkey, other poultry)?** (*Tick one box*)									
☐Never	☐ Less than once a month	☐1-3 times per month	☐Once a week	☐2-4 times per week	☐5-6 times per week	☐Once a day	☐2-3 times per day	☐4-5 times per day	☐Every time I eat

**How often do you eat red meat (e.g. beef, pork, veal, lamb)?** (*Tick one box*)									
☐Never	☐ Less than once a month	☐1-3 times per month	☐Once a week	☐2-4 times per week	☐5-6 times per week	☐Once a day	☐2-3 times per day	☐4-5 times per day	☐Every time I eat

**How often do you eat processed meat (e.g. cured ham and turkey, salami)? (Tick one box)**									
☐Never	☐ Less than once a month	☐1-3 times per month	☐Once a week	☐2-4 times per week	☐5-6 times per week	☐Once a day	☐2-3 times per day	☐4-5 times per day	☐Every time I eat

**How often is the meat and processed meat you eat certified organic?***** (*Tick one box*)					
☐Always	☐Often	☐Sometimes	☐Rarely	☐Never	☐I don't know

**How often do you eat fish or shellfish, including processed fish (e.g. canned tuna, smoked salmon)?** (*Tick one box*)									
☐Never	☐ Less than once a month	☐1-3 times per month	☐Once a week	☐2-4 times per week	☐5-6 times per week	☐Once a day	☐2-3 times per day	☐4-5 times per day	☐Every time I eat

**How often is the fish and shellfish you eat certified organic?*** (*Tick one box*)					
☐Always	☐Often	☐Sometimes	☐Rarely	☐Never	☐I don't know

**How often do you eat/drink dairy products (excluding cheese) (e.g. milk, yogurt, buttermilk, kefir , skyr and other fermented dairy products)?** (*Tick one box*)									
☐Never	☐ Less than once a month	☐1-3 times per month	☐Once a week	☐2-4 times per week	☐5-6 times per week	☐Once a day	☐2-3 times per day	☐4-5 times per day	☐Every time I eat

**How often are the dairy products you eat certified organic?*** (*Tick one box*)					
☐Always	☐Often	☐Sometimes	☐Rarely	☐Never	☐I don't know

**What kind of milk/plant-based milk substitutes do you mainly drink?** (*Tick one box*)				
☐Whole fat milk (e.g. cow, goat, sheep)	☐ Semi-skimmed milk (e.g. cow, goat, sheep)	☐Skimmed milk (e.g. cow, goat, sheep)	☐Plant based milk substitutes (e.g. oat, pea, almond, soy, coconut)	☐None of these

**How often is the milk** (cow, goat, sheep, etc.) **you drink certified organic?*** (*Tick one box*)					
☐Always	☐Often	☐Sometimes	☐Rarely	☐Never	☐I don't know

**How often are the plant-based milk substitutes you drink certified organic?*** (*Tick one box*)					
☐Always	☐Often	☐Sometimes	☐Rarely	☐Never	☐I don't know

**How often do you eat cheese?** (*Tick one box*)									
☐Never	☐ Less than once a month	☐1-3 times per month	☐Once a week	☐2-4 times per week	☐5-6 times per week	☐Once a day	☐2-3 times per day	☐4-5 times per day	☐Every time I eat

**How often is the cheese you eat certified organic?*** (*Tick one box*)					
☐Always	☐Often	☐Sometimes	☐Rarely	☐Never	☐I don't know

**How often do you eat butter and/or margarine?** (*Tick one box*)

<table>
<tr><td>☐Never</td><td>☐ Less than once a month</td><td>☐1-3 times per month</td><td>☐Once a week</td><td>☐2-4 times per week</td><td>☐5-6 times per week</td><td>☐Once a day</td><td>☐2-3 times per day</td><td>☐4-5 times per day</td><td>☐Every time I eat</td></tr>
<tr><td colspan="10">How often is the butter and/or margarine you eat certified organic?*** (Tick one box)</td></tr>
<tr><td colspan="2">☐Always</td><td colspan="2">☐Often</td><td colspan="2">☐Sometimes</td><td colspan="2">☐Rarely</td><td>☐Never</td><td>☐I don't know</td></tr>
<tr><td colspan="10">How often do you eat eggs? (Tick one box)</td></tr>
<tr><td>☐Never</td><td>☐ Less than once a month</td><td>☐1-3 times per month</td><td>☐Once a week</td><td>☐2-4 times per week</td><td>☐5-6 times per week</td><td>☐Once a day</td><td>☐2-3 times per day</td><td>☐4-5 times per day</td><td>☐Every time I eat</td></tr>
<tr><td colspan="10">How often are the eggs you eat certified organic?*** (Tick one box)</td></tr>
<tr><td colspan="2">☐Always</td><td colspan="2">☐Often</td><td colspan="2">☐Sometimes</td><td colspan="2">☐Rarely</td><td>☐Never</td><td>☐I don't know</td></tr>
<tr><td colspan="10">How often do you drink alcohol (e.g. wine, beer)? (Tick one box)</td></tr>
<tr><td>☐Never</td><td>☐ Less than once a month</td><td>☐1-3 times per month</td><td>☐Once a week</td><td>☐2-4 times per week</td><td>☐5-6 times per week</td><td>☐Once a day</td><td>☐2-3 times per day</td><td>☐4-5 times per day</td><td>☐Every time I eat</td></tr>
<tr><td colspan="10">What kind of alcohol do you mainly drink? (Tick one box)</td></tr>
<tr><td colspan="3">☐Wine</td><td colspan="3">☐Beer</td><td colspan="2">☐Spirits (e.g. vodka, gin, rum and similar)</td><td colspan="2">☐Other:</td></tr>
<tr><td colspan="10">How often are the alcoholic beverages you eat certified organic?*** (Tick one box)</td></tr>
<tr><td colspan="2">☐Always</td><td colspan="2">☐Often</td><td colspan="2">☐Sometimes</td><td colspan="2">☐Rarely</td><td>☐Never</td><td>☐I don't know</td></tr>
<tr><td colspan="10">How often do you drink sugary drinks (e.g. coke, orange soda) including fruit juices and sugar sweetened tea and coffee? (Tick one box)</td></tr>
<tr><td>☐Never</td><td>☐ Less than once a month</td><td>☐1-3 times per month</td><td>☐Once a week</td><td>☐2-4 times per week</td><td>☐5-6 times per week</td><td>☐Once a day</td><td>☐2-3 times per day</td><td>☐4-5 times per day</td><td>☐Every time I eat</td></tr>
</table>

How often are the sugary drinks you eat certified organic?*** (*Tick one box*)

☐Always	☐Often	☐Sometimes	☐Rarely	☐Never	☐I don't know

How often do you eat fast food (e.g. burgers, shawarma, hot dog or similar)? (*Tick one box*)

☐Never	☐ Less than once a month	☐1-3 times per month	☐Once a week	☐2-4 times per week	☐5-6 times per week	☐Once a day	☐2-3 times per day	☐4-5 times per day	☐Every time I eat

How often is the fast food you eat certified organic?*** (*Tick one box*)

☐Always	☐Often	☐Sometimes	☐Rarely	☐Never	☐I don't know

How often do you eat desserts/sweets (e.g. ice cream, cake)? (Tick one box)

☐Never	☐ Less than once a month	☐1-3 times per month	☐Once a week	☐2-4 times per week	☐5-6 times per week	☐Once a day	☐2-3 times per day	☐4-5 times per day	☐Every time I eat

How often is the dessert/sweets you eat certified organic?*** (*Tick one box*)

☐Always	☐Often	☐Sometimes	☐Rarely	☐Never	☐I don't know

How often do you eat sauce (incl. hot and cold sauces, e.g. béchamel, béarnaise sauce and ketchup, mayonnaise, cocktail sauce) (*Tick one box*)

☐Never	☐ Less than once a month	☐1-3 times per month	☐Once a week	☐2-4 times per week	☐5-6 times per week	☐Once a day	☐2-3 times per day	☐4-5 times per day	☐Every time I eat

How often is the sauce you eat certified organic?*** (*Tick one box*)

☐Always	☐Often	☐Sometimes	☐Rarely	☐Never	☐I don't know

How often do you eat processed salty snacks (e.g. nuts, crisps, popcorn, crackers, pretzels)? (*Tick one box*)

☐Never	☐ Less than	☐1-3 times	☐Once a week	☐2-4 times	☐5-6 times	☐Once a day	☐2-3 times per day	☐4-5 times	☐Every time I eat

	once a month	per month		per week	per week			per day	

**How often are the salty snacks you eat certified organic?***** (*Tick one box*)					
☐Always	☐Often	☐Sometimes	☐Rarely	☐Never	☐I don't know

**What kind of oil/fat do you mainly use to cook and flavour?** (*Tick maximum 3 boxes*)			
☐Olive oil	☐Butter	☐Rapeseed oil	☐Sunflower oil
☐Ghee	☐Corn oil	☐Flaxseed oil	☐Sesame oil
☐Coconut oil	☐Vegan butter	☐Other: ____	

**Do you follow a certain diet?** (*Tick all that apply*)				
☐Vegan (Plant based diet. Do not eat any animal products or by-products)	☐Lacto-ovo-vegetarian (Mainly plant based diet. Do eat dairy products and egg products. Do not eat red or white meat, fish or shellfish)	☐Lacto-vegetarian (Mainly plant based diet Do eat dairy products, e.g. cheese, milk and yoghurt. Do not eat red or white meat, fish or shellfish and eggs)	☐Ovo-vegetarian (Mainly plant based diet. Do eat egg products. Do not eat red or white meat, fish or shellfishand dairy products)	☐Flexitarian/semi-vegetarian diet (Mainly plant based diet. Do occasionally eat fish and/or meat)
☐ Pesco-vegetarian (Mainly plant based diet. Do not eat red or white meat , but do eat	☐Lactose free (Do not eat milk and milk products)	☐Gluten free (Do not eat food that contains the gluten, including wheat, rye and barley)	☐Mediterranean (Do eat fish and shellfish, whole grains, fruits, vegetables, pulses, herbs, spices, nuts and healthy fats such as olive oil as core foods)	☐Paleo (Do not eat processed foods, grains and sugar)

fish and shellfish)				
☐ No	☐ Other			

**How important are the attributes listed below for your food choices? Please, rate.** *(grading from 1 – not important at all, to 5 – very important).*	
**Attributes**	**Grading**
Price	
Taste	
Appearance	
Safety (e.g. pathogens, pesticide residue)	
Sustainable packaging (e.g. biodegradable, reusable, non-plastic)	
Composition (ingredients)	
Nutritional value	
Origin (I prefer local products)	
Without genetically modified organisms (GMOs)	
Certificates	
Seasonality	
Freshness	
Naturalness (no artificial food additives)	
**How important are the aspects listed below when you think about "sustainable" food? Please, rate.** (*grading from 1 – not important at all, to 5 – very important*).	
Low environmental impact	
Availability and affordability of food for all	
No use of pesticides and genetically modified organisms (GMOs)	
Locally produced	

Minimally processed				
Healthy				
Organic				
Traditional				
Plant-based/vegetarian food				
Animal welfare				
Fair revenue for farmers				
**Would you like to change your food habits into more sustainable diets?**				
☐Yes	☐No	☐I don't know		
If yes, **what are you ready to change?***(Tick maximum 3 boxes)*				
☐Eat more certified organic foods	☐Eat more seasonal fruits and vegetables	☐Eat less meat and more plant-based/vegetarian food	☐Eat more locally produced foods	☐Eat less dairy
☐Spend more money on foods for which farmers get a fair price	☐Waste less foods at home	☐Choose foods produced with high animal welfare standards	☐Spend more money on sustainable foods	☐Other:________________________
If no, **what are the main reasons preventing you from eating (more) sustainably?** *(Tick maximum 3 boxes)*				
☐Lack of information on how to do so	☐Lack of clear labelling	☐I'm not concerned with sustainability	☐Lack of sustainable food products in my usual shopping / eating places	☐Too expensive
☐Lack of time (to buy	☐Appearance and taste	☐It would not be in accordance with my	☐I don't believe my behaviour/choices/food	☐Other: ________________________

it, to cook it, etc.)		friends/other household members needs	habits can make any difference	
**Do you obtain food from other sources than supermarkets such as farmer's market, food box schemes, community-supported-agriculture (CSA)?******				
☐Yes		☐No		
If yes, **how often do you purchase your food from such alternative initiatives?**				
☐Sometimes	☐Often	☐Always	☐ I Don't know	
**In which of the initiatives and alternative places listed below do you obtain your food. Please, tick the box(es) of your choice:**				
☐ Community-Supported Agriculture (CSA)	☐Food box schemes	☐Farmer's markets	☐Specialised organic shops	☐Directly from the farm
☐From consumer cooperatives	☐In online shops	☐Own (home) breeding/cultivation	☐Other: ____________________________	
**Thank you very much for completing this questionnaire. In case of <u>any comments,</u> you can type these in the space below.**				

Table S2. Frequency of choosing organic products by household survey respondents in Warsaw (% of respondents).

Product Category	Always (%)	Often (%)	Sometimes (%)	Rarely (%)	Never (%)	I don't know (%)
Fruits and dried fruits	4	17	24	18	9	28
Vegetables	2	15	29	24	9	22
Legumes	3	11	19	19	20	29
Nuts	2	9	17	21	23	29
Bread	3	7	14	19	27	30
Cereal products	2	10	18	22	17	31
Potatoes	6	10	11	14	30	29
Meat	3	9	16	18	29	25
Fish and shellfish	3	9	14	19	27	30
Dairy products	4	12	19	19	21	24
Milk	4	8	6	12	52	19
Plant-based drink	8	4	8	4	78	3
Cheese	2	8	17	20	23	29
Butter and/or margarine	3	6	12	15	37	28
Eggs	29	18	14	11	14	13
Alcoholic beverages	2	3	6	15	41	33
Sugary drink	1	2	5	10	55	28
Fast food	0	1	2	5	50	42
Desserts/sweets	1	4	8	18	31	38
Sauce	2	4	9	14	35	37
Salty snacks	1	2	8	14	39	36

Table S3. Answers to the household survey question: "How important are the following attributes for your food choices?"

	1 - not important at all	2	3	4	5 - very important
Price (%)	2	12	31	30	24
Taste (%)	0	1	5	23	71
Appearance (%)	4	8	27	3	25
Safety (%)	2	7	16	29	45
Sustainable packaging (%)	6	12	31	35	16
Composition (ingredients) (%)	1	2	10	27	60
Nutritional value (%)	3	6	21	33	36
Origin (I prefer local products) (%)	7	12	31	31	20
Without GMO (%)	15	16	22	22	25
Certificates (%)	14	23	36	19	8
Seasonality (%)	3	7	23	40	27
Freshness (%)	0	0	2	21	76
Naturalness (%)	1	2	14	31	52
